# Combining a computerized cognitive test with serum biomarkers improves detection of early-onset neurodegenerative disorders

**DOI:** 10.1177/13872877251389224

**Published:** 2025-10-27

**Authors:** Veera Tikkanen, Teemu I Paajanen, Anna-Leena Heikkinen, Eino Solje, Kasper Katisko, Tarja Kokkola, Sari Kärkkäinen, Sanna-Kaisa Herukka, Nadine Huber, Annakaisa Haapasalo, Tuomo Hänninen, Christer Hublin, Anne M Koivisto, Jussi Virkkala, Toni T Saari, Anne M Portaankorva, Johanna Krüger

**Affiliations:** 1 Research Unit of Clinical Medicine, Neurology, University of Oulu, Oulu, Finland; 2 Medical Research Center Oulu, Oulu University Hospital, Oulu, Finland; 3 Work Ability and Working Careers Unit, Finnish Institute of Occupational Health, Helsinki, Finland; 4 Neurocenter, Neurology, Oulu University Hospital, Oulu, Finland; 5 Neurocenter, Neurology, Kuopio University Hospital, Kuopio, Finland; 6 Neurology, Institute of Clinical Medicine, University of Eastern Finland, Kuopio, Finland; 7 A.I. Virtanen Institute for Molecular Sciences, University of Eastern Finland, Kuopio, Finland; 8 Occupational Medicine and Work Ability, Finnish Institute of Occupational Health, Helsinki, Finland; 9 Department of Geriatrics and Neurological Memory Clinic, Helsinki University Hospital, Helsinki, Finland; 10 Clinical Neurosciences, University of Helsinki, Helsinki, Finland; 11 Clinical Neurophysiology and Clinical Neurosciences, HUS Diagnostic Center, University of Helsinki and Helsinki University Hospital, Helsinki, Finland; 12 Institute for Molecular Medicine Finland (FIMM), University of Helsinki, Helsinki, Finland

**Keywords:** Alzheimer's disease, blood-based biomarkers, digital cognitive assessment, early-onset dementia, glial fibrillary acidic protein, mild cognitive impairment, neurofilament light chain, neuropsychological tests

## Abstract

**Background:**

Early diagnosis of early-onset dementia (EOD) is often challenging. Executive dysfunction is a common symptom in EOD, including Alzheimer's disease (AD). Currently, no specific tools for screening EOD at the primary health care exist. The availability of neuropsychological assessment and biomarker analyses is often limited to specialized memory clinics. However, recent advances in blood-biomarkers and better accessible computerized cognitive tests provide new opportunities.

**Objective:**

To investigate the ability of serum biomarkers neurofilament light chain and glial fibrillary acidic protein, combined with a novel computerized Flexible Attention Test (FAT) to distinguish early-onset neurodegenerative disorders from other conditions resulting cognitive symptoms in a diagnostically challenging memory clinic population.

**Methods:**

Cohort consisted of 206 participants with symptom onset ≤65 years and followed up to 24 months: EOD (n = 54, including 29 AD cases), neurological mild cognitive impairment (MCI-n, n = 34), MCI due to other causes (n = 104), and subjective cognitive decline (n = 14). Serum biomarkers were analyzed using single-molecule array, and discriminative accuracy of individual and combined tests was assessed using Receiver Operating Characteristic and discriminant analyses. FAT was compared to traditional neuropsychological tests.

**Results:**

Combining serum biomarkers with cognitive tests were more accurate in detecting EOD and MCI-n from other conditions (area under the curve, AUC = 0.872) compared to each method individually (AUC = 0.633–0.783). Combination of the FAT and serum biomarkers reached 82.1% accuracy, comparable to traditional neuropsychological tests and biomarkers together (82.3%).

**Conclusions:**

Integrating serum biomarkers with computerized FAT offers a promising strategy for screening EOD early and identifying patients for further evaluation.

## Introduction

The rising global prevalence of cognitive disorders poses a significant public health challenge.^1^ Age is the most significant risk factor for neurodegenerative disorders, but disease variants affecting younger age-groups are also possible. Alzheimer's disease (AD) is the most common subtype of early-onset dementia (EOD),^2^ defined as dementia with symptom onset at or before 65 years of age. Early diagnostics of EOD can be particularly challenging due to the frequent presence of atypical symptom presentation,^3^ substantial symptom overlap between different subtypes, and a high risk of misdiagnosis, often as psychiatric disorders.^4^ These factors can lead to delayed diagnosis and significant distress for patients and their families. Furthermore, atypical variants of AD are especially common in younger individuals, making early recognition and accurate diagnosis even more difficult.^5^ Additionally, factors such as burnout, sleep disturbances, and non-progressive neurological conditions can lead to cognitive complaints or even mild cognitive impairment (MCI).^6–8^ Some individuals are ultimately classified as having subjective cognitive decline (SCD) after thorough evaluation shows no objective deficits.^9^ Early differentiation of non-progressive causes of cognitive symptoms at the primary care level is essential to avoid unnecessary referrals and to reduce the economic burden on specialized health care services.

Recent studies have focused on blood-based biomarkers, such as neurofilament light chain (NfL) and glial fibrillary acidic protein (GFAP), which are found at elevated levels during neuroinflammation and axonal injury and can be measured using ultrasensitive assays.^10,11^ Elevated levels of NfL and GFAP have been linked to various neurological conditions^12–14^ and, to a lesser extent, psychiatric disorders.^15^ While promising in discriminating AD, MCI, frontotemporal dementia and healthy controls,^16–18^ as well as neurodegenerative disorders from psychiatric disorders,^19^ these markers are not disease-specific. Furthermore, their association with cognitive performance has produced mixed findings,^20^ possibly due to limited cognitive profiling in prior studies.

Neuropsychological assessment is important for EOD diagnostics, especially given that in many forms of EOD, early cognitive symptoms manifest as problems with executive functions (EFs) rather than episodic memory loss.^3^ Dysexecutive symptoms are often subtle and not readily captured by standard cognitive screening tools, and unlike episodic memory deficits, which are typically more apparent in brief evaluations, executive dysfunction requires more specialized testing for accurate detection and differential diagnosis. Several theoretical models have been proposed to define and categorize EFs. One widely accepted framework distinguishes three core components: set shifting, working memory updating, and inhibition.^21^ However, EFs are also frequently conceptualized more broadly, often overlapping with constructs such as attention, metacognition, problem-solving, and self-regulation. For example, some models embed EFs within working memory systems as functions of the central executive.^22^ Recent studies have increasingly focused on the use of computer-based tests for assessing cognitive performance. Previously, we demonstrated that the computer-based Flexible Attention Test (FAT), which evaluates EFs, attention, and visuospatial working memory, is a promising tool for distinguishing patients with EOD from those experiencing cognitive problems due to other causes.^23^ The FAT battery comprises eight subtasks assessing attention, visuomotor speed, cognitive flexibility, and visuospatial working memory. It has shown potential in differentiating EOD from other causes of cognitive impairment^23^ and in detecting even very mild cognitive deficits associated with covert cerebral small vessel disease.^24^ The advantages of digital cognitive tests are that they are easy to use and provide improved stimulus presentation, measurement precision, and timing accuracy,^25^ making them promising tools especially for the screening of neurodegenerative disorders.

The aim of this study was to investigate how serum NfL (sNfL) and GFAP (sGFAP) measurements, combined with the novel computerized FAT battery can differentiate EOD and MCI due to a neurological etiology (MCI-n) from other causes of MCI (MCI-o) and SCD in memory clinic patients under the age of 65. These results were compared with the combination of the same serum biomarkers and cognitive performance in traditional neuropsychological assessment. We also examined correlations between serum biomarkers and cognitive performance, and studied which combination best identifies EOD and MCI-n patients from other patient groups.

## Methods

### Participants

Altogether 210 patients were recruited as part of the *Cognitive Impairment and Work Ability* (CIWA) study between March 2019 and March 2021 at their first visit to a specialized memory outpatient clinic at the Oulu and Kuopio University Hospitals in Finland. All patients included in the study had been referred to diagnostic investigations due to a suspected neurodegenerative disorder, and they had the onset of symptoms no later than at the age of 65. 206 patients had at least one measurement of either sNfL or sGFAP and neuropsychological data available forming the study population in this paper. The study and data management were coordinated by The Finnish Institute of Occupational Health.

This study was performed in line with the principles of the Declaration of Helsinki. Approval was granted by the Ethics Committees of the Northern Ostrobothnia Hospital and Northern Savo Hospital districts.

### Diagnostic outcomes

**Figure 1.**
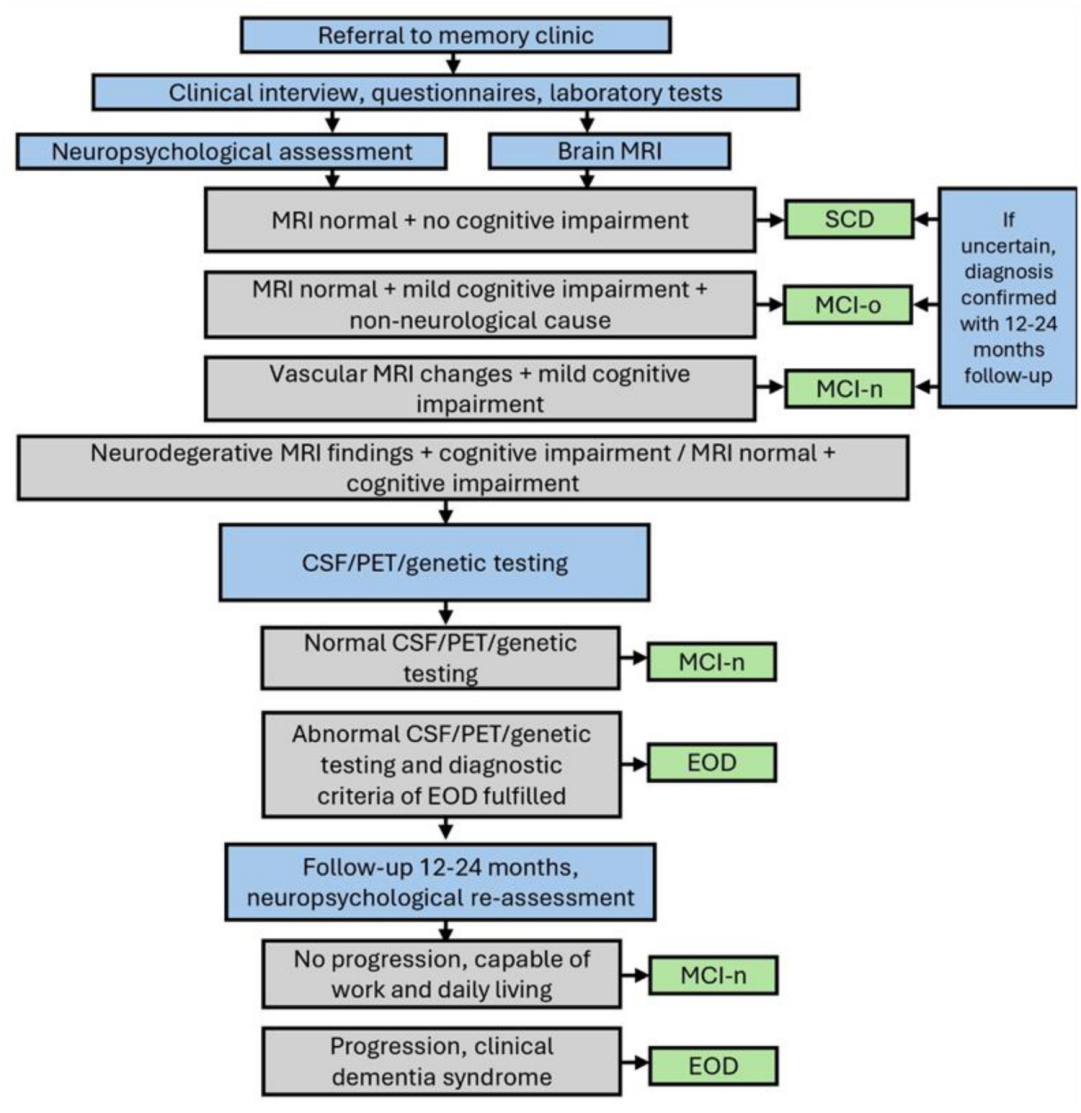
Expert-driven clinical decision pathway used to classify patients into diagnostic groups. Diagnostic classification was based on comprehensive clinical evaluation and followed international consensus criteria.

**Figure 2.**
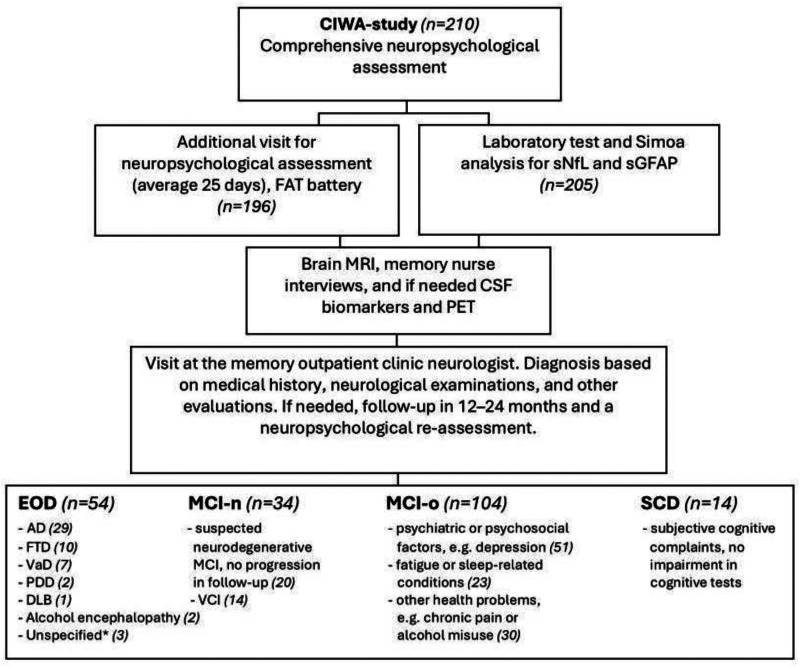
The Cognitive Impairment and Work Ability (CIWA) study participants. FAT: Flexible Attention Test; sNfL: serum neurofilament light; sGFAP: serum glial fibrillary acidic protein; MRI: magnetic resonance imaging; CSF: cerebrospinal fluid; PET: positron emission tomography; EOD: early-onset dementia; AD: Alzheimer's disease; FTD: frontotemporal dementia; VaD: vascular dementia; PDD: Parkinson's disease dementia; DLB: dementia with Lewy bodies; MCI-n: mild cognitive impairment due to neurological reasons; MCI-o: mild cognitive impairment due to other reasons; VCI: vascular cognitive impairment; SCD: subjective cognitive decline. *Three patients were diagnosed with dementia due to a neurodegenerative disorder; however, the exact subtype could not be determined by the time of their final visit during this study.

Patients were diagnosed according to current diagnostic criteria^26–31^ by neurologists specialized in neurodegenerative diseases, using a comprehensive, multidisciplinary evaluation including medical history, neurological and neuropsychological assessments, laboratory tests, and brain structural magnetic resonance imaging (Figure 1). When required for clinical diagnosis, cerebrospinal fluid analysis (including amyloid-β_42_, tau, and phosphorylated tau, n = 81), genetic testing (n = 7), and fluorodeoxyglucose positron emission tomography (n = 12) were also utilized. In unclear cases, symptom progression was followed for up to two years. According to the follow up information, patients were classified into four groups (Figure 2): EOD (N = 54), MCI-n (N = 34; possible neurodegenerative or vascular etiology without progression), MCI-o (N = 104; impairment due to psychiatric, sleep-related, or other non-neurodegenerative causes), and SCD (N = 14; subjective complaints without objective impairment or identifiable cause).

### Neuropsychological assessment and formulation of domain scores

A comprehensive neuropsychological assessment and an additional study-specific cognitive test session were conducted, as previously detailed.^32^ A variety of neuropsychological tests were performed to evaluate key domains of cognitive functions. Raw test scores were converted into z-scores using the SCD group as a reference. When necessary, the scales were inverted so that higher values represented better performance in all variables. Domain scores were calculated by averaging z-scores of individual tests. For domains consisting of 2 or 3 tests, one missing value was permitted, whereas for domains consisting of 4 or 5 tests, up to two missing values were allowed. Table 1 shows all the variables used for the present analysis and their classification into eight cognitive domains (Executive Function, Attention and Vigilance, Working Memory, Verbal Memory, Visual Memory, Language Skills, Visuospatial Skills, and Processing Speed). These included common neuropsychological tests, but also less commonly used but clinically useful tests. The computerized touch screen-based FAT battery was developed by Dr Paajanen and programmed by Dr Virkkala at the Finnish Institute of Occupational Health. It consists of eight short subtasks measuring visuomotor speed, attention, set shifting and visual working memory, and is described in detail in previous publications.^23,24^ We calculated z-scores also for the FAT subtasks using the SCD group as the reference and created two compound scores: one for subtasks measuring visuomotor speed, attention and EFs (subtasks 1–6, named FAT Executive), and another for visuospatial working memory (subtasks 7–8, named FAT Working Memory). In the first score, up to two missing values were allowed, while the visuospatial memory score required at least one completed test.

**Table 1.** The neuropsychological test battery used in the cognitive impairment and work ability (CIWA) study.

Cognitive domain	Neuropsychological tests used in the assessment
Executive Function	Trail Making Test Part B ^33^, Stroop color-word subtest (50 items),^34^ Phonemic Fluency (P/A/S),^35^ Alternating S,^36^ dual task (combined phonemic fluency K and alternating S)^23^
Attention and Vigilance	247 Cancellation test,^35^ Stroop color subtest (50 items)^34^
Processing Speed	Trail Making Test Part A,^33^ Digit Symbol Coding and Symbol Search (Wechsler Adult Intelligence Scale IV)^37^
Working Memory	Digit Span and Letter-Number Sequencing (Wechsler Adult Intelligence Scale IV)^37^
Verbal Memory	Logical Memory I and II, Word List I and II and recognition task (Wechsler Memory Scale III)^38^
Visual Memory	Visual Reproduction I and II (Wechsler Memory Scale III),^38^ Rey-Osterrieth Complex Figure Test immediate and delayed recall^35^
Language Skills	Similarities and Comprehension (Wechsler Adult Intelligence Scale IV),^37^ Semantic fluency,^35^ 15-Item version of the Boston Naming Test^39^
Visuospatial Skills	Block Design and Visual Puzzles (Wechsler Adult Intelligence Scale IV),^37^ Rey-Osterrieth Complex Figure Test copy^35^
FAT Executive	Reaction time, Numbers, Numbers and Letters, Numbers and Shapes, Numbers and Months Forward, Number and Months Backward (Flexible Attention Test)^23^
FAT Working Memory	Memory Forward, Memory Backward (Flexible Attention Test)^23^

### Snfl and sGFAP analyses

Serum samples were processed, frozen, and stored according to standardized procedures. The levels of sNfL and sGFAP were measured at the Biomarker laboratory of the University of Eastern Finland. Quantification was performed using the Single Molecule Array (Simoa) digital immunoassays and the Quanterix Simoa HD-X analyzer (Quanterix, Billerica, MA, USA) following the manufacturer's instructions.^11^ The sGFAP levels were analyzed with the Simoa GFAP Discovery Kit (Quanterix ref. 102336), and sNfL levels with the Simoa NfL Advantage Kit (Quanterix ref. 103186). All analyses were performed by operators blinded to clinical data.

### Statistical analyses

Group differences in gender, education level, and work status were analyzed using the chi-square test, and differences in age, sNfL and sGFAP levels, and neuropsychological test performance with the non-parametric Kruskal-Wallis test. Bonferroni correction was applied for multiple comparisons. Spearman's correlation coefficient was used for correlation analyses. Receiver operating characteristic (ROC) analysis was used to evaluate group discrimination, providing the area under the curve (AUC), sensitivity, and specificity values. Combined biomarker and neuropsychological performance was assessed using logistic regression–based predicted probabilities, which were included in ROC analysis. AUC differences were tested using the DeLong's test,^40^ and cut-offs were determined via the Youden Index.^41^ Stepwise discriminant analyses were conducted including neuropsychological domains, FAT scores, sNfL, sGFAP, and age, to assess the ability of various combinations of these variables to discriminate either the EOD and MCI-n patients or the EOD patients alone from the other groups. Due to shape of the variable distributions, sNfL and sGFAP values were log-transformed to improve reliability in analyses. A p-value < 0.05 was considered significant. All analyses were conducted using IBM SPSS Statistics version 29.0.

## Results

### Demographics

The demographic characteristics of all participants, and divided within four diagnostic groups, are presented in Table 2. No statistically significant differences were found between the diagnostic groups in terms of gender (p = 0.902) or education level (p = 0.549). However, the EOD and MCI-n patients were older (on average 4.8 years) (p < 0.001) compared to the MCI-o and SCD patients. Additionally, a significant difference in employment status (p < 0.001) was identified. The SCD patients were more frequently employed than the EOD or MCI-n patients. Furthermore, the EOD patients were significantly more likely to be outside the workforce compared to the MCI-o patients (p < 0.001).

**Table 2.** Demographics and clinical characteristics.

	Total sample N = 206	Diagnostic outcome				
		EOD N = 54	MCI-n N = 34	MCI-o N = 104	SCD N = 14	p
Age (y), mean (SD)	57.6 (6.1)	60.1 (4.6)	60.7 (5.2)	55.5 (6.3)	55.5 (5.0)	<0.001^a,b,c,d^
Gender, %Female	56.8	53.7	55.9	57.7	64.3	0.902
Education, %:						
≤ 9 y	16.7	18.5	15.2	17.5	7.1	0.549
10–12 y	52.9	53.7	63.6	50.5	42.9	
>12 y	30.4	27.8	21.2	32.0	50.0	
Working, % (versus sick-leave, unemployed or retired)	38.3	18.5	32.4	45.2	78.6	<0.001^a,b,d^

EOD: early-onset dementia; MCI-n: mild cognitive impairment-neurological; MCI-o: mild cognitive impairment-other; SCD: subjective cognitive decline. Significant (p < 0.05) pairwise group comparisons after correction with the Bonferroni method for multiple testing: ^a^EOD versus MCI-o; ^b^EOD versus SCD; ^c^MCI-n versus MCI-o; ^d^MCI-n versus SCD.

### Group differences in sNfL, sGFAP, and cognitive performance

The neuropsychological performance, along with sNfL and sGFAP concentrations across the four diagnostic groups, is summarized in Table 3. The levels of sNfL and sGFAP were significantly higher in the EOD patients compared with the SCD group (p ≤ 0.008) and with the MCI-o group (p < 0.001). Additionally, the EOD patients had higher sGFAP concentrations than the MCI-n patients (p = 0.043), while no significant difference was observed for sNfL (p = 0.088). However, sNfL concentration was statistically significantly higher in the MCI-n patients compared to the MCI-o patients (p = 0.005), while there was no difference in the sGFAP concentrations between these groups (p = 0.064).

**Table 3.** Serum GFAP and NfL and neuropsychological performance results for the total sample and the diagnostic groups.

	Total sample (N = 206)	Mean (standard deviation)				
		EOD (N = 54)	MCI-n (N = 34)	MCI-o (N = 104)	SCD (N = 14)	P
sNfL(pg/mL)	15.0_4_ (16.4)	24.8 (28.0)	14.6_1_ (7.7)	10.5_3_ (5.7)	11.1 (4.9)	<0.001^b^^,c,d^
sGFAP (pg/mL)	143.0_2_ (84.2)	207.5_1_ (102.7)	150.3 (83.4)	112.4_1_ (53.5)	105.5 (47.0)	<0.001^a,b,c^
Executive Function	−1.8_6_ (2.5)	−3.3_2_ (3.3)	−1.8_2_ (1.9)	−1.2_2_ (1.9)	0.0 (0.6)	<0.001^b,c,e,f^
Attention and Vigilance	−1.2_1_ (2.3)	−2.6_1_ (2.9)	−1.0 (1.4)	−0.7 (1.9)	0.0 (0.7)	<0.001^b,c^
Working Memory	−1.0_3_ (1.6)	−1.8_1_ (1.7)	−1.1_2_ (1.3)	−0.8 (1.6)	0.0 (0.8)	<0.001^b,c,e^
Verbal Memory	−1.4_3_ (1.4)	−2.4 (1.3)	−1.2_2_ (1.2)	−1.1_1_ (1.1)	0.0 (0.7)	<0.001^a,b,c,e,f^
Visual Memory	−1.8_5_ (1.4)	−2.7_1_ (1.4)	−2.0_2_ (1.1)	−1.5_2_ (1.2)	0.0 (0.7)	<0.001^b,c,e,f^
Language Skills	−1.5 (1.4)	−2.1 (1.5)	−1.7 (1.1)	−1.3 (1.4)	0.0 (0.7)	<0.001^b,c,e,f^
Visuospatial Skills	−2.0 (2.4)	−3.5 (3.6)	−1.9 (1.5)	−1.5 (1.6)	0.0 (0.5)	<0.001^b,c,e,f^
Processing Speed	−0.9_3_ (1.2)	−1.9_1_ (1.3)	−1.0_1_ (0.8)	−0.6_1_ (1.0)	0.0 (0.9)	<0.001^b,c,e^
FAT Executive	−1.9_12_ (3.8)	−4.4_6_ (5.6)	−1.9 (2.7)	−1.0_6_ (2.7)	0.0 (0.8)	<0.001^b,c,e^
FAT Working Memory	−0.4_16_ (1.0)	−0.9_9_ (1.0)	−0.6_1_ (1.0)	−0.2_6_ (1.0)	0.0 (1.0)	0.010^b^

Neuropsychological results are presented in z scores based on the SCD group performance level and variation. EOD: early-onset dementia; MCI-n: mild cognitive impairment-neurological; MCI-o: mild cognitive impairment-other; SCD: subjective cognitive decline; FAT: Flexible Attention Test. Significant (p < 0.05) pairwise group comparisons after correction with the Bonferroni method for multiple testing: ^a^EOD versus MCI-n; ^b^EOD versus MCI-o; ^c^EOD versus SCD; ^d^MCI-n versus MCI-o; ^e^MCI-n versus SCD; ^f^MCI-o versus SCD. The numbers in the subscript (e.g., x_1_) represent the missing values.

The performance in all neuropsychological domains and FAT Executive score was significantly weaker in the EOD patients compared with the MCI-o and SCD patients (p < 0.001 in both). A closer examination of group differences in neuropsychological performance revealed that most neuropsychological domains showed statistically significant differences between the MCI-n and SCD groups. Several domains also showed significant differences between the MCI-o and SCD groups at the group level. In contrast, only the verbal memory domain showed a statistically significant difference between the EOD and MCI-n groups. Altogether, there were very few missing values among the analyzed parameters (Table 3). For serum biomarkers, missing values were due to failed laboratory sample analysis.

### Correlations between sNfL, sGFAP, and neuropsychological test scores

Statistically significant, although generally weak, negative correlations were observed between sNfL, sGFAP, traditional neuropsychological domain scores, and FAT compound scores. For sNfL, correlations with neuropsychological domains ranged from r = −0.18 to −0.38 (p < 0.05 in all), with the strongest associations observed for processing speed (r = −0.38), FAT Executive compound scores (r = −0.37), and verbal and visual memory (r = −0.28 and r = −0.27, respectively). sGFAP showed similar but slightly weaker correlations (r = −0.11 to −0.28), with processing speed (r = −0.28), FAT Executive (r = −0.28), and visual memory (r = −0.25) showing the strongest associations. All correlations were statistically significant (p < 0.05), except the correlation between language skills and sGFAP (r = −0.11, p = 0.106). The correlation between sNfL and sGFAP levels was moderate (r = 0.55, p < 0.001).

### ROC analyses

**Figure 3.**
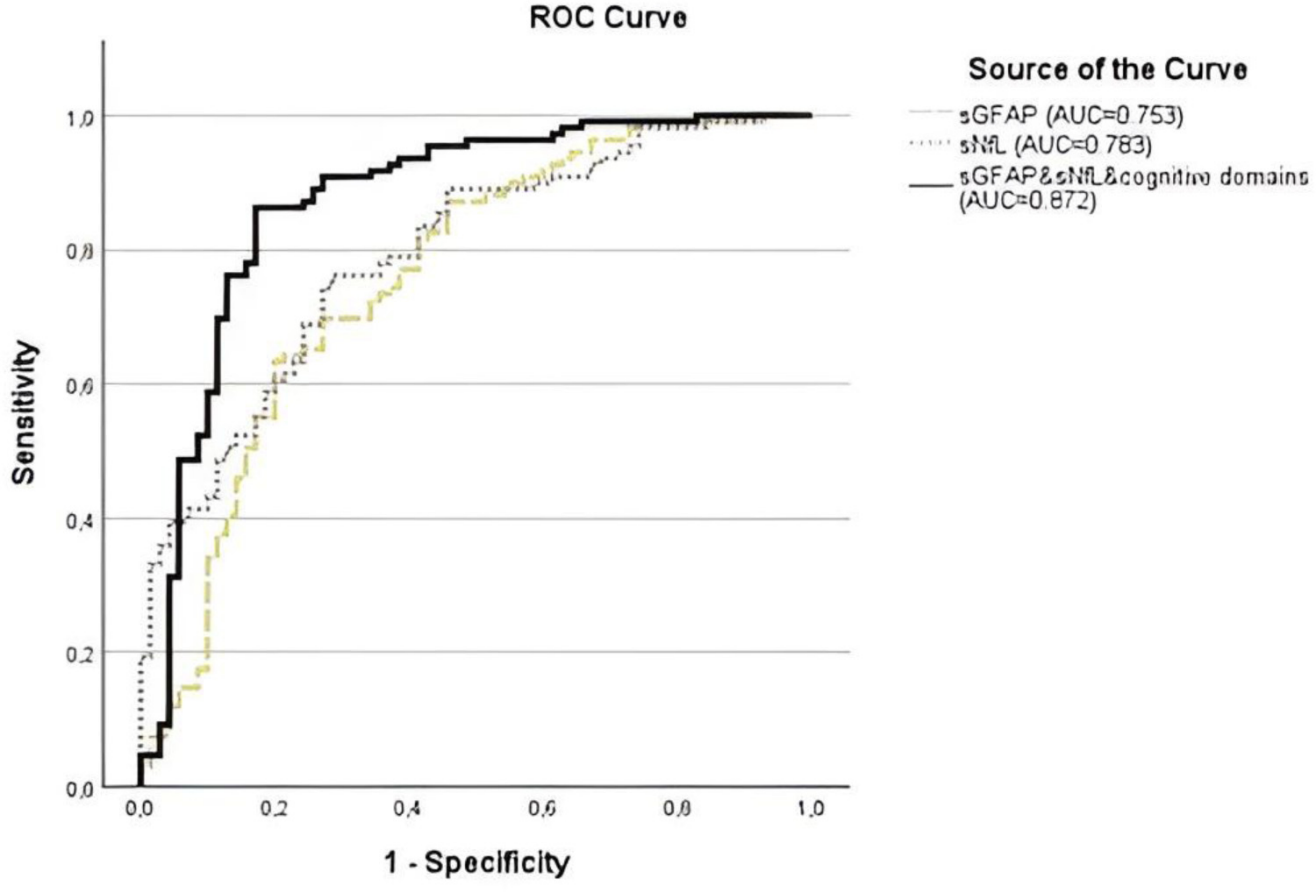
Serum neurofilament light (sNfL), serum glial fibrillary acidic protein (sGFAP), and the predicted probability of the combination (sNfL, sGFAP, all neuropsychological domains, and flexible attention test combination scores) model receiver operating characteristic (ROC) curves in comparing early-onset dementia (EOD) and mild cognitive impairment-neurological (MCI-n) versus mild cognitive impairment-other (MCI-o) and subjective cognitive decline (SCD).

We investigated the ability of sNfL and sGFAP to distinguish the EOD and MCI-n patients from the MCI-o and SCD patients (Figure 3). Both sNfL (AUC = 0.783, 95% CI 0.721–0.845, p < 0.001) and sGFAP (AUC = 0.753, 95% CI 0.685–0.822, p < 0.001) showed moderate accuracy in differentiating the groups. A cut-off of 11.8 pg/mL for sNfL yielded 70.1% sensitivity and 73.9% specificity, while 113.1 pg/mL for sGFAP resulted in 79.3% sensitivity and 61.5% specificity.

When analyzing the ability of the neuropsychological domains and FAT combination scores to distinguish the EOD and MCI-n patients from the MCI-o and SCD patients, the FAT Executive score demonstrated stronger accuracy (AUC = 0.756, 95% CI 0.688–0.824, p < 0.001), whereas the FAT Working Memory score was weaker but still moderate (AUC = 0.633, 95% CI 0.554–0.713, p = 0.002). The accuracies of the neuropsychological domains were at least moderate: Executive Function (AUC = 0.715, 95% CI 0.643–0.787, p < 0.001), Attention and Vigilance (AUC = 0.699, 95% CI 0.626–0.773, p < 0.001), Working Memory (AUC = 0.661, 95% CI 0.585–0.737, p < 0.001), Verbal Memory (AUC = 0.724, 95% CI 0.650–0.797, p < 0.001), Visual Memory (AUC = 0.735, 95% CI 0.664–0.806, p < 0.001), Language Skills (AUC = 0.672, 95% CI 0.598–0.746, p < 0.001), Visuospatial Skills (AUC = 0.665, 95% CI 0.590–0.740, p < 0.001), and Processing Speed (AUC = 0.766, 95% CI 0.701–0.831, p < 0.001).

Using logistic regression analysis, we constructed a combined variable that, included all neuropsychological domain variables, the FAT combination scores, sNfL, and sGFAP. The predicted probability of belonging to the diagnostic group (EOD and MCI-n versus*.* others) showed good discrimination accuracy (AUC = 0.872, 95% CI 0.814–0.931, p < 0.001) (Figure 3). This combined variable performed significantly better than sGFAP (p = 0.001), sNfL (p = 0.007), and any of the neuropsychological domains or the FAT combination scores alone (p ≤ 0.005 in all).

### Discriminant function analyses

Using stepwise discriminant analysis, we identified the most effective combination of variables for distinguishing the EOD and MCI-n patients from the MCI-o and SCD patients (Table 4). The first model included age, all eight cognitive domains based on the traditional neuropsychological assessment, sNfL, and sGFAP. The highest classification accuracy (82.3%) in this model was achieved with a combination of sNfL, sGFAP, the visual memory domain, and the processing speed domain (sensitivity 84.2%, specificity 79.8%). In the second model when the FAT compound scores were included alongside sNfL, sGFAP, and age, the most discriminative variables were age, sNfL, sGFAP and the FAT Executive score. This model yielded discrimination accuracy of 82.1% (sensitivity 81.8%, specificity 82.5%).

**Table 4.** Stepwise discriminant analysis (a) early-onset dementia (EOD) and mild cognitive impairment-neurological (MCI-n) versus other groups (b) EOD versus other groups.

Variables candidate for the model	Variables included in the model	% correctly classified	% sensitivity	% specificity
Age, sNfL, sGFAP, EF, Att, WM, VM, VisM, LS, VS, PS	a) sNfL, sGFAP, VM, PS b) sNfL, sGFAP, VM, PS	a) 82.3 b) 82.8	a) 84.2 b) 84.9	a) 79.8 b) 82.1
Age, sNfL, sGFAP, FAT Executive, FAT WorkMem	a) Age, sNfL, sGFAP, FAT Executive b) sNfL, sGFAP, FAT Executive	a) 82.1 b) 84.2	a) 81.8 b) 85.1	a) 82.5 b) 83.9
Age, sNfL, sGFAP	a) Age, sNfL, sGFAP b) sNfL, sGFAP	a) 76.5 b) 79.0	a) 74.6 b) 77.4	a) 79.1 b) 79.6
Age, EF, Att, WM, VM, VisM, LS, VS, PS, FAT Executive, FAT WorkMem	a) Age, VM, PS b) VM, PS	a) 78.9 b) 78.4	a) 78.0 b) 75.9	a) 80.2 b) 79.3

EF: executive function; Att: attention and vigilance; WM: working memory; VM: verbal memory; VisM: visual memory; LS: language skills; VS: visuospatial skills; PS: processing speed; FAT: Flexible Attention Test; WorkMem: Working Memory score in the FAT battery.

When focusing specifically on distinguishing the EOD patients from all other groups, the classification accuracy improved slightly. The best-performing model combined the FAT Executive score with serum biomarkers, correctly classifying 84.2% of cases (Table 4).

Overall, combining neuropsychological variables (the traditional neuropsychological domains or the FAT scores) with serum biomarkers, the models correctly classified the EOD and MCI-n patients from the MCI-o and SCD patients with an accuracy of 82.1–82.3%. Additionally, they distinguished the EOD patients from all other groups with an accuracy of 82.8–84.2%. Models based solely on neuropsychological measures correctly classified 78.4–78.9% of cases, while those relying only on serum biomarkers achieved 76.5–79.0% accuracy.

## Discussion

In this study, the aim was to evaluate whether combining serum biomarker levels with a short computerized cognitive FAT test could distinguish the patients with EOD and MCI-n from the patients with cognitive problems due to potentially reversible etiology or SCD. Our results show that the combination of FAT battery and serum biomarkers measurements provides similar or even slightly better discriminative accuracy as compared to traditional neuropsychological assessment domain scores combined with biomarkers data. This is a promising finding, especially given the highly challenging differential diagnostic setting in memory clinic populations.

As expected, AD emerged as the largest single diagnostic group within the EOD population. However, the AD group was not analyzed separately, as the primary focus of this study was to explore tools that could help on the early identification of individuals who are likely to have a progressive neurodegenerative condition and therefore should be referred for more detailed diagnostic evaluation, rather than aiming for precise differential diagnosis.

Our findings align with previous studies showing elevated sNfL and sGFAP levels in dementia patients compared to those with psychiatric disorders or subjective cognitive concerns.^16–18^ These biomarkers appeared to differentiate dementia patients from healthy controls more clearly than they differentiated patients with mood, sleep, or stress-related disorders from controls. However, it is important to note that the mood disorder patients in this study were referred to a memory clinic assessment rather than to psychiatric evaluation and thus did not represent severe psychiatric illness.

As expected, neuropsychological performance significantly differed across diagnostic groups, with the EOD patients performing worse on average in nearly all the tests compared to the MCI-o and SCD patients. This is consistent with previous findings showing deficits in EFs, memory, and processing speed in EOD.^42,43^

Notably, the FAT battery results correlated with sNfL and sGFAP levels at least as strongly as the traditional neuropsychological assessment results. However, all the correlations between the sNfL, sGFAP, and neuropsychological variables were relatively weak. The strength of these associations is likely affected, at least in part, by individual differences in serum biomarker levels^44^ and cognitive performance.^45^ Additionally, the heterogeneity of our patient population may have contributed to the weaker correlations.^20^ If healthy controls had been included, it is possible that the correlations would have been stronger. Among the FAT subtasks, especially the numbers, number–letter tasks, and visuospatial span have previously been found to be more strongly associated with white matter hyperintensities than many conventional paper-and-pencil tests.^24^

Cognitive impairment in younger patients have diverse causes, and not all individuals referred to memory outpatient clinics are diagnosed with a memory disorder. In clinical practice, distinguishing patients with definite or suspected neurodegenerative disease (EOD, MCI-n groups) from those with cognitive symptoms due to potentially reversible causes (MCI-o, SCD groups) is essential, as the former group requires further diagnostic evaluation in special health care. To address this, we examined the ability of serum biomarkers, traditional neuropsychological domains, and the FAT combination scores to differentiate the EOD and MCI-n patients from the MCI-o and SCD groups. All these measures showed mainly moderate discriminative ability. Interestingly, sNfL and sGFAP levels and their optimal cut-offs were lower than in many previous studies, likely due to differences in patient demographics: our sample included only individuals under 65, whereas biomarker levels such as NfL are known to exponentially increase with age.^46^ Assay-related variability may also contribute to discrepancies across studies, highlighting the importance of standardization in the future.^10,47^ Despite this, our results showed relatively high classification accuracy, supporting earlier findings that blood-based NfL may be more informative in younger than older individuals.^48^ However, specificity values remained relatively low, highlighting the limitations of relying on a single biomarker for diagnostic purposes. Combining sNfL and sGFAP improved diagnostic accuracy compared to using either biomarker alone, supporting the view that they reflect distinct yet interconnected neurobiological processes.^49^ Additionally, the moderate correlation between sNfL and sGFAP aligned with previous findings in healthy population.^50^

In this study, we focused on the combined use of sNfL, sGFAP, and comprehensive set of neuropsychological measures including both traditional methods and computerized test. As expected, integrating serum biomarkers and neuropsychological data improved statistically significantly the ability to identify patients in the EOD and MCI-n groups compared to using either approach alone. This finding aligns with previous research showing that combining NfL data with the Boston Naming Test enhanced the diagnostic accuracy of AD.^51^ When considering which combination best identifies EOD and neurological MCI patients from others, the most effective combination included the verbal memory domain and the processing speed domain for the traditional neuropsychological tests with sNfL and sGFAP measurements (82.3% correctly classified). While EF deficit is known to be a key feature of early-stage EOD,^3^ this finding emphasizes that memory and processing speed should also be assessed. Given the complex, multidimensional nature of EF, it is likely that EF difficulties manifest in other cognitive domains as well.^52^ The combination of FAT Executive score, sNfL and sGFAP data, and age was equally effective (82.1% correctly classified). Interestingly, the combination of FAT Executive score and sNfL and sGFAP data yielded even slightly higher accuracy (84.2%) than the combination of traditional neuropsychological tests and serum biomarker data (82.8%) when only EOD was distinguished from the other groups. Considering that the tablet-based, self-administrable FAT battery is easier and cheaper to provide even in primary health care settings compared to a comprehensive neuropsychological assessment, this finding is clinically relevant. It suggests that digital tools assessing executive functions and processing speed could usefully complement, or even enhance, traditional assessments in future diagnostic protocols. They may provide new possibilities for screening patients in primary health care who should be referred for further examinations at a memory outpatient clinic.

One of the strengths of our study is its heterogeneous patient population, which reflects the diverse cases and diagnostic challenges encountered in real world memory outpatient clinic setting. This increases the ecological validity of our findings and supports their applicability in everyday clinical practice. However, we acknowledge that such heterogeneity may also be considered a limitation, as it introduces variability that can complicate statistical interpretation and reduce internal consistency. Additionally, our neuropsychological assessment was comprehensive, covering a broader range of cognitive functions than many previous studies. Another strength of our study is the long follow-up period, with patients being followed for up to 24 months when necessary to confirm the correct diagnosis.

This study also has some limitations. The subgroup sample sizes were relatively small, particularly in the SCD group. Moreover, the group sizes for individual neurodegenerative diseases were too small to allow separate analyses. In addition, healthy controls were not available for this study. Instead, the SCD group served as a reference, which better reflects the clinical reality and offers clinically relevant comparison in the context of early-phase cognitive assessment.

In conclusion, our findings suggest that combining the computerized FAT battery with serum biomarkers sNfL and sGFAP data is an effective and clinically applicable approach for detecting EOD and MCI during the initial phase of clinical evaluation. The FAT battery, which is short and easily self-administrable, demonstrated at least comparable accuracy to traditional neuropsychological tests when combined with biomarker measurements, supporting its use. As recognizing the patients with early phase EOD timely remains challenging, particularly in primary healthcare settings, there is a need for accessible and effective methods to reliably identify potential neurodegenerative disorder patients. By integrating cognitive and biological markers, a multimodal approach enhances diagnostic precision and outperforms reliance on single biomarkers. Future studies should validate these findings in larger cohorts, including healthy controls, and explore the potential of additional screening methods to further improve diagnostic accuracy.

## Data Availability

The data supporting the findings of this study are available on request from the corresponding author. The data are not publicly available due to privacy or ethical restrictions.
